# Recent developments in visible light induced polymerization towards its application to nanopores

**DOI:** 10.1039/d2cc06595a

**Published:** 2023-01-19

**Authors:** Claire Förster, Annette Andrieu-Brunsen

**Affiliations:** a Macromolecular Chemistry – Smart Membranes, Technische Universität Darmstadt 64287 Darmstadt Germany annette.andrieu-brunsen@.tu-darmstadt.de

## Abstract

Visible light induced polymerizations are a strongly emerging field in recent years. Besides the often mild reaction conditions, visible light offers advantages of spatial and temporal control over chain growth, which makes visible light ideal for functionalization of surfaces and more specifically of nanoscale pores. Current challenges in nanopore functionalization include, in particular, local and highly controlled polymer functionalizations. Using spatially limited light sources such as lasers or near field modes for light-induced polymer functionalization is envisioned to allow local functionalization of nanopores and thereby improve nanoporous material performance. These light sources are usually providing visible light while classical photopolymerizations are mostly based on UV-irradiation. In this review, we highlight developments in visible light induced polymerizations and especially in visible light induced controlled polymerizations as well as their potential for nanopore functionalization. Existing examples of visible light induced polymerizations in nanopores are emphasized.

## Abstract 2

This review summarizes the state of the art of visible light induced polymerizations and highlights their potential for nanopore functionalization.

## Graphical abstract

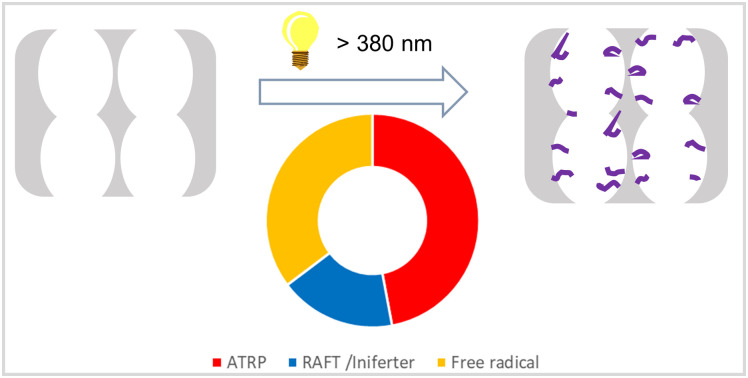

## Introduction

Polymer functionalization of nanoscale pores advanced in the last years. This advance has been driven by the resulting material properties but as well by new possibilities in the field of polymerization itself, as *e.g.* the increasing number of reported visible light induced polymerizations.^1–3^ Polymer functionalized nanopores, for example, allow for specific control of molecular transport which is relevant in separation, energy conversion, sensing, or release.^2,4–10^ Especially, gating of molecular transport using stimuli-responsive polymers in nanopores has been demonstrated for all possible stimuli from pH, ion interaction to redox or even magnetic gating.^7,11^ However, for nanopore transport beyond gating, controlled- and local (polymer-) functionalization is one essential tool aiming to control polymer amount, chain composition, and local gradient formation for various monomers. Such asymmetric design of porous materials has been shown to result in Janus materials for side selective separation or increased transport performance.^3,12^ For example, theoretical studies from Huang and Szleifer demonstrated the relevance of controlled and local nanopore functionalization, as well as polymer sequence design of nanopores allowing transport direction.^13,14^

An experimental implementation of local polymer functionalization including polymer sequence design has to be based on controlled (living) polymerizations. The general concept of controlled (living) polymerizations was first realized for anionic polymerizations in 1956.^15^ A more robust way to control polymer functionalization tolerating a large variety of different monomers can be realized by controlled radical polymerizations. Often-used controlled radical polymerizations are atom transfer radical polymerization (ATRP),^16,17^ reversible addition fragmentation chain transfer polymerization (RAFT),^18^ nitroxide-mediated radical polymerization^19,20^ (NMP) and iniferter initiated polymerization.^21–23^ In recent years these polymerizations were increasingly often initiated using visible light irradiation including spatial and temporal control over chain growth for example at planar surfaces. Furthermore, these polymerizations allow for mild reaction conditions, since the use of light often eliminates the need for high temperatures.^24^

Although, most polymerization initiators absorb UV-light, visible light, here defined for wavelength between 380–750 nm, is an interesting trigger. Among others, it is cheap, abundant in nature, and lasers usually operate in the visible wavelength range.^25^ Furthermore, initiation by visible light is often considered to be sustainable as it allows to use sunlight and bears some aspects of green chemistry as it is abundant in nature.^26–28^ Thereby, both, catalyst-free approaches and polymerization with the use of photo catalysts were realized, to date.^29–31^

Besides visible light irradiation, also high throughput processes are an emerging field of research, in context of polymer synthesis.^32–35^ In particular visible light can be used for realization of high throughput processes.^33,36,37^ Data driven material design needs large data and sample sets as shown for data driven nanopore application in the context of sensing.^3,38^ Thus, automated synthesis is essential and of potentially increasing importance in future. This renders visible light induced polymerization with a potential for automated process design interesting.

In this review different visible light induced polymerization techniques are discussed in more detail, focusing on their potential for nanopore functionalization and especially for (nano)local and automated polymer nanopore functionalization. In addition, especially examples for visible light induced polymerizations in nanopores are highlighted.

## Free radical polymerizations initiated by visible light

Visible light induced free radical polymerizations receive increasing attention within the last decade. In general, two classes of photoinitiators are used for visible light induced, free radical polymerizations: Type I photoinitiators generate radicals by irradiation to a single-molecule bond cleavage. Type II photoinitiators generate radicals by interaction with a second molecule due to a bimolecular reaction after irradiation.^39,40^ For visible light irradiation mostly type II photoinitiators are used.^39^

But especially in the last decade also type I photoinitiators were developed for visible light induced polymerizations.^41^ For example He *et al.*^42^ recently used the type I photoinitiator dimethyl 1,4-dibenzoylformate (DM-BD-F) under 405 nm irradiation to polymerize acrylate monomers. But also wavelength up to 600 nm were used for visible light induced polymerizations initiated by type I photoinitiators, as demonstrated by the group of Haas using triacylstannenolates as initiator.^43^ Currently, especially the broadening of the applicable wavelengths range, water solubility, automated fabrication technology as well as absorption and bleaching behaviour of type I photoinitiators for visible light induced polymerizations are investigated.^41^ In the following two aspects are highlighted in more detail: dye-sensitized polymerizations which allow to use a broad wavelength range as well as to polymerize in water and the application of visible-light induced free radical polymerizations for additive manufacturing as automated fabrication method.

**Fig. 1:**
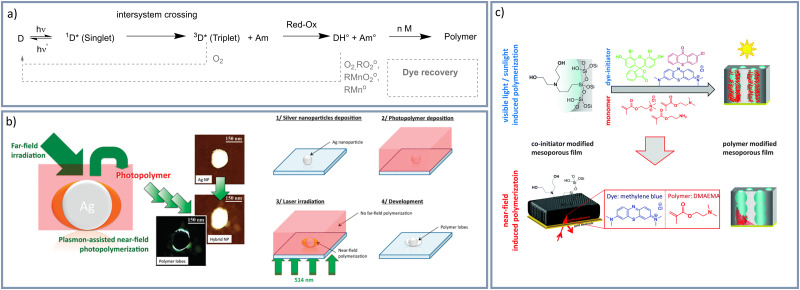
(a) Photopolymerization by dye (d)-amine (Am)-System.^45^ Adapted with permission from *J. Am. Chem. Soc.*, 2011, **133**, 10535–10542.^45^ Copyright 2021 American Chemical Society. (b) Plasmon-assisted near field photopolymerization by Deeb *et al.*^45^ Reprinted (adapted) with permission from *J. Am. Chem. Soc.*, 2011, **133**, 10535–10542.^45^ Copyright 2021 American Chemical Society. (c) Plasmon near-field induced surface polymer modification of mesoporous thin silica films.^48^ Reprinted with permission *Chem. Commun.*, 2015, **51**, 11697–11700.^48^ Copyright 2015 Royal Society of Chemistry.

With respect to broadening the wavelength range and allowing polymerization in water an interesting free radical visible light induced polymerization, based on type II photoinitiators, is the so-called dye-sensitized photopolymerization. Dye-sensitized polymerizations allow polymerization at basically the entire visible light wavelength range depending on the selected dye photosensitizer absorption. Lalevèe and Fouassier summarized trends in dye sensitized radical polymerizations in their book “Dyes and Chromophores in Polymer Science” (2015).^44^ Briefly, by using two-component photo initiating systems (Fig. 1a),^45^ consisting of a photosensitizer (dye) and a coinitiator (*e.g.* a tertiary amine) polymerizations using various dyes and thus at different wavelengths are accessible.^46^ For example cyanine dyes together with irradiation at a wavelength range of 510–570 nm^47^ were addressed. While using methylene blue red light with a wavelength around 660 nm was used to initiate this radical polymerization.^48^ But also sunlight was used for polymerization using various dyes.^49^ Deeb *et al.*^45^ performed a nanoscale dye-sensitized visible-light induced photopolymerization using surface plasmons generated by silver nanoparticles (Fig. 1b). They show nanoscale polymer formation in the areas of surface plasmon enhancement by selecting an irradiation energy below a so-called threshold energy. In further work, it was demonstrated that polymerization is indeed initiated *via* the photochemical mechanism and not by local temperature increase or hot electrons under the applied low irradiation energy.^50^ Our research group^48,51,52^ applied dye-sensitized visible-light induced photopolymerizations using a dye-tertiary amine photosensitizer, photoinitiator combination for mesoporous silica functionalization resulting in polymer-dominated gating of ionic mesopore accessibility. By using surface plasmons localized polymer functionalization in mesoporous layers was achieved (Fig. 1c).^48^ Besides using visible light and implementing local control on polymer placement, automation of polymerization is of relevance especially in data-driven material design approaches which need large data sets. Automation of local polymer structuring using visible light induced free radical polymerizations was realized using direct laser writing (DLW) in combination with photoresists. This combination let to the development of additive manufacturing of three dimensional polymer materials.^53–55^ The combination of visible light induced, free radical polymerization with 3D printing is a rapidly growing research area.^41,56,57^ For example, Lalevèe and colleagues^58^ used ketone derivatives for a free radical polymerization under 405 nm light and demonstrated 3D printing. Breloy *et al.*^46^ used methacrylated quinizarin derivatives to achieve complex 3D biosourced structures under 405 nm light irradiation for 3D-photoprinting technology. Also using 405 nm, Zhao and colleagues^59^ used dealkaline lignin for digital light processing 3D printing. Zhang *et al.*^60^ used blue, green, yellow, and red LEDs to initiate a free radical polymerization and furthermore, demonstrated 3D printing by using a polychromatic visible light (400–730 nm) 3D printer. In nanoporous materials DLW or 3D printing in combination with free radical polymerizations have not been demonstrated to date.

## Visible light induced ATRP

Besides, the above mentioned free radical polymerization ATRP, as one of the most widely used methods of controlled radical polymerization,^16,61^ was applied to surface polymer grafting using porous silica in 1997 by Huang and Wirth.^62^ In 2012 Kruk^63^ summarized polymer functionalization of mesoporous silica using ATRP. For example, Fu *et al.*^64^ reported the adjustment of pore sizes in mesoporous silica particles due to increasing polymer amount using ATRP. The group of Schönherr^65^ recently analyzed the confinement effects in anodic alumina nanopores and demonstrated the effect of curvature and pore diameter effect on the polymerization kinetics using SI-ATRP. ATRP was as well used to functionalize mesoporous silica^66–70^ and ion track etched pores^71–73^ with different polyelectrolytes to study *e.g.* the resulting permselective ion transport in such polyelectrolyte functionalized mesoporous thin films. In the last years research has been directed, among others, towards visible light induced ATRP. The majority of studies focuses on solution polymerization. Thereby often small amounts of metal catalysts have been used. But also metal-free visible light induced ATRPs based entirely on organic catalysts have been reported. Since 2014 photoinduced organocatalyzed ATRP gained increasing interest.^74^ Konkolewicz *et al.*^75^ used a few ppm of copper catalyst for a photoinduced ATRP under blue and violet LED irradiation and even sunlight whereby at 631 nm no successful ATRP at was observed. Also in the presence of ppm copper catalysts Ciftci *et al.*^76^ managed a visible light induced ATRP using Mn_2_(CO)_10_ as photocatalyst. Pan *et al.*^77^ described an ATRP, which was induced at 392 nm. Upon 380 nm irradiation, a controlled radical polymerization using Ir(ppy)_3_ (ppy = 2-pyridylphenyl) as photoredoxcatalyst was reported by Treat *et al*.^78^ Because of the robust nature of the catalyst the polymerization allows even monomers with acid groups to be polymerized. Ir(ppy)_3_ as photoredoxcatalyst was as well applied by the group of Zhu^79^ performing a visible light induced ATRP. Zhu *et al.* managed to separate and recycle the catalyst after polymerization. Besides metal organic complexes, organic photocatalysts were used for visible light induced ATRPs. Matyjaszewski and coworkers^80^ reported a green light induced ATRP using a combination of Eosin Y and a copper catalyst to achieve ATRP in contact with air. In 2014 visible light induced, metal free ATRPs were reported by the groups of Hawker^81^ and Theriot.^82^ But already in 2012 the group of Yagci^83^ described the control of molecular weight and distribution using bis (2,4,6-trimethylbenzoyl) phenylphosphine oxide (BAPO), Eosin Y, and Erythrosin B at 400–500 nm light for ATRP. Liu *et al.*^84^ used fluorescein as organocatalyst, the group of Yagci^85^ used reducible dyes and amine as well as alkylhalides, and Wang *et al.*^86^ described an ATRP with an organic semiconductor based visible light activated photo catalyst. In 2018 Xu *et al.*^87^ demonstrated the suitability of metal-free, visible light induced ATRP for the polymerization of acrylamides. Very recently Qiao *et al.*^88^ used carbon quantum dots as catalyst for a visible light induced ATRP at 405 nm. Using these carbon quantum dots a high monomer conversion of more than 90% in one minute was achieved, making polymerization suitable for 3D printing.^88^ Although most of the visible light induced ATRP were performed at wavelengths below 600 nm, there have been studies on ATRP using 630 nm^89^ or even using near infrared irradiation.^90^

**Fig. 2:**
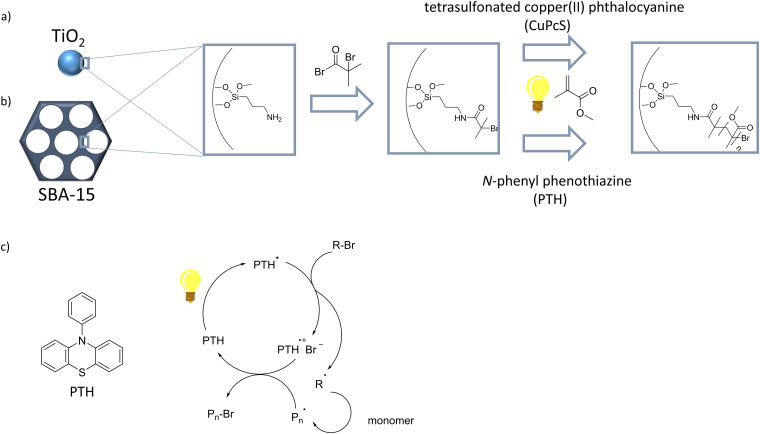
(a) SI-ATRP under visible light irradiation on TiO_2_ particles using CuPcS as photo catalyst by Bansal *et al.*^91^ Adapted with the permission from *RSC Adv.*, 2015, **5**, 21189–21196.^91^ Copyright 2015 Royal society of chemistry. (b) SI-ATRP under visible light irradiation on SBA-15 using PTH as photo catalyst by Ma *et al.*^98^ Adapted with permission from *Polymers*, 2017, **9**, 58.^98^ Copyright 2017 by the authors; licensee MDPI, Basel, Switzerland. (c) Mechanism of the metal free photo imitated ATRP using PTH as photo catalyst by the group of Yagci.^99^ Reprinted with the permission from *Polym. Chem.*, 2016, **7**, 6039–6043.^99^ Copyright 2016 Royal society of chemistry.

These initial developments on visible light induced ATRP in solution were also transferred to surface grafting of polymers using visible light induced ATRPs. Bansal *et al.*^91^ reported a surface initiated ATRP (SI-ATRP) under visible light irradiation using tetrasulfonated copper(ii) phthalocyanine (CuPcS) as catalyst (Fig. 2a). Under visible light irradiation CuPcS is reduced to Cu(i) complex by a one-electron transfer process.^91^ To achieve polymer grafting in a first step Bansal *et al.*^91^ functionalized TiO_2_-particels with (3-aminopropyl)triethoxysilane and 2-bromoisobutyryl bromide, which was used as initiator for the surface initiated polymerization. The polymerization was then carried out using visible light irradiation and a varying irradiation time of 5–24 h at room temperature. Also on surfaces Jiang *et al.* prepared self-healing nanocomposite hydrogels using a SI-PET (photoinduced electron/energy transfer)-ATRP under visible light irradiation for 15 h.^92^ Fan *et al.*^93^ also performed a SI-PET-ATRP to prepare self-healing nanocomposite hydrogels. As in solution, on surfaces and in pores metal free ATRPs are carried out in recent years. The group of Hawker^94^ reported a metal free SI-ATRP under visible light at 405 nm and room temperature. They used *N*-phenyl phenothiazine (PTH) as catalyst and an irradiation time from 0.5–4 h. The group of Matyjaszewski^95^ used PTH as photocatalyst while functionalizing 16 nm and 120 nm silica nanoparticles with poly(methyl methacrylate) using a wavelength of 365 nm. Also using irradiation of 365 nm and PTH as catalyst SI-ATRP's to functionalize mesoporous silica polymer nanocomposites^96^ and SBA-15^97^ were reported. A visible light induced SI-ATRP in mesoporous silica was as well achieved by Ma *et al.*^98^ while using 380 nm light to functionalize SBA-15 (Fig. 2b). The group of Yagci^99^ analyzed the photoinitiation mechanism of photoinduced metal free ATRP using PTH (Fig. 2c).

Also performing a metal-free SI-ATRP on SBA-15 the group of Zhang^100,101^ used fluorescein as photo catalyst and triethylamine as electron donor under blue light irradiation. Another example of pore functionalization using visible light induced ATRP was performed by Meng *et al.*,^102^ while using the photo catalyst *fac*-[Ir(ppy)_3_] they functionalized microporous polypropylene membrane (MPPM) surfaces with methacrylates and diblock copolymer brushes. Local polymer-reinitiation at the nanoscale has recently been demonstrated by Soppera and Colleagues^103^ using a visible light induced ATRP and surface plasmons at 532 nm.

Numerous visible light induced ATRP's have been performed at different wavelengths, both with and without metal catalysts. The listed examples clearly show that visible light induced ATRP is well studied in solution, and that it is increasingly studied on surfaces, while it becomes recently a research topic also in porous materials.

## Visible light induced RAFT and Iniferter initiated polymerizations

Besides ATRP, RAFT is a versatile controlled radical polymerization mechanism as well applied to porous materials and in recent years combined with visible light irradiation. The classical RAFT polymerization was reported in 1988 by the Commonwealth Scientific and Industrial Research Organization (CSIRO) research group.^18^ One advantage of RAFT polymerization is the large variety of different monomers which are compatible with the RAFT mechanism. Because of the high tolerance to different functional groups the polymerization can even be performed in aqueous solution. The RAFT process uses thiocarbonylthio-compounds to control the polymerization. Basis of the polymerization is the reversible degenerative chain transfer.^104^ The mechanism of RAFT consists mainly of two reactions: the pre-equilibrium and the main equilibrium.

RAFT agents show characteristic colors, which indicate their absorbance in the visible light region such as the yellow trithiocarbonates and dithiobenzoates being red. When the RAFT agents are exposed to suitable light, carbon-centered and sulfur-centered radicals are generated. The carbon-centered radicals are usually responsible for initiating the polymerization.^105^ The photolytic stability of various RAFT agents under blue light was analyzed by the group of Qiao.^106^ They reported that the stability of the RAFT agent (*i.e.* thiocarbonylthio-compounds) depends on the structure of the fragmenting group (*R*-). The reactivity of the carbon-centered radical depends on the photolytic cleavage.^106^

Light induced RAFT is usually realized either by the PET^107^ or the iniferter (initiator–transferagent–terminator^21^) approach.^108^ Thereby, PET-RAFT is a visible light induced variant of the RAFT polymerization, which was developed in 2014 by the group of Boyer.^107^ Interestingly, this PET-RAFT provides oxygen tolerance under ambient conditions and can be performed either using a transition metal-based or an organic photoredox catalyst such as *e.g.* Ru(bpy)_3_Cl_2_, ZnTPP or Eosin Y, respectively.^109^ The alternative approach of iniferter initiated polymerization using RAFT agents to polymerize upon visible light irradiation were first investigated by Otsu.^110^ Already in 1958 Otsu and Nayatani^111^ used thiuram disulfides to polymerize styrol in solution discussing two types of iniferters: an asymmetric A–B type and a symmetric C–C type iniferter,^110,112^ where the A–B type is preferred for better reactivity control, and *e.g.* preparation of block-*co*-polymers.^110^ For further details on iniferter initiated polymerizations we recommend the article by Otsu.^110^

As already observed for ATRP a significant number of visible light induced RAFT and iniferter initiated polymerizations were performed in solution and on planar surfaces. Especially the relatively recently reported PET-RAFT seems to be promising for controlled visible light induced polymer functionalization. Depending on the required wavelength, different catalysts can be used, both on metal and organic basis. In comparison to PET-RAFT polymerization, which can be initiated up to 850 nm,^113^ iniferter initiated polymerizations are usually initiated at a wavelength below 400 nm. Whereas, in recent years iniferter initiated polymerizations using visible light were also described. On the other hand, iniferter initiated polymerization don’t require any additional photo catalyst, which might be advantageous in the context of nanopore polymer functionalization with respect to reagent availability in these nanopores during polymerization.

**Fig. 3:**
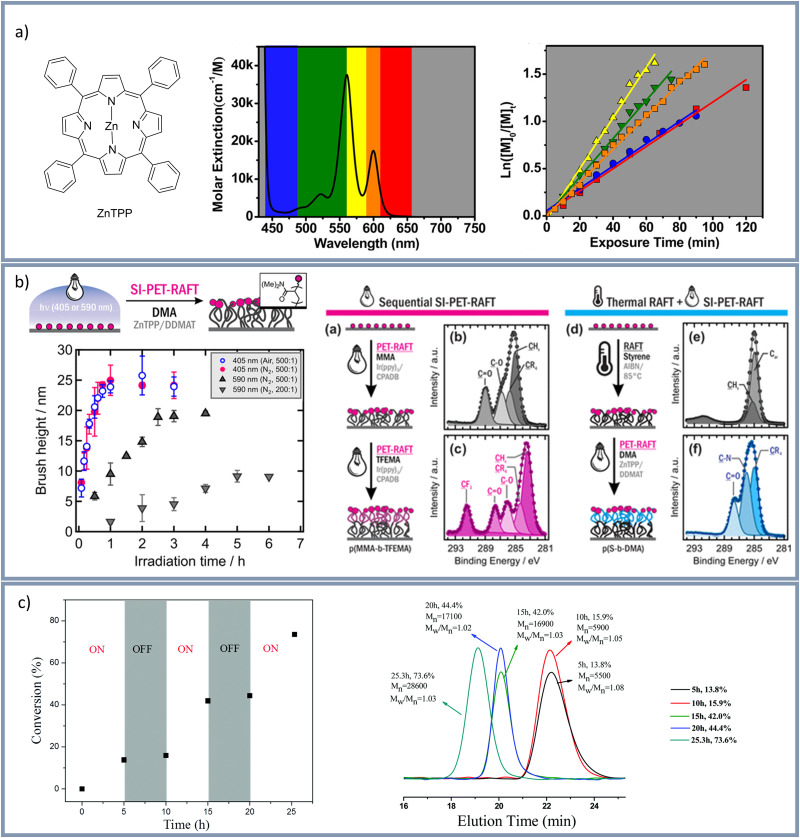
(a) Molecular structure of the photo catalyst ZnTPP (left). Molar extinction spectrum of ZnTPP with the corresponding LED colors (middle) and plot of ln([*M*]_0_/[*M*]_t_) *vs.* exposure time depending on different light wavelengths (right) by Shanmugam *et al.*^120^ Reprinted with the permission from *J. Am. Chem. Soc.*, 2015, **137**, 9174–9185.^120^ Copyright 2015 American Chemical Society. (b) Surface-initiated PET-RAFT by group of Pester^109^ Polymer brush thickness *vs.* irradiation time under inert gas and under air and diblock copolymer synthesis by SI-PET-RAFT. Reprinted with the permission from *ACS Macro Lett.*, 2019, **8**, 374–380.^109^ Copyright 2019 American Chemical Society. (c) Visible light mediated RAFT by the group of Pan^121^ using PTH as photo catalyst, demonstrating “ON/OFF” control over polymerization. Reproduced with the permission from *Mater. Chem. Front.*, 2017, **1**, 1200–1206^121^ with permission from the Chinese Chemical Society (CCS), Institute of Chemistry of Chinese Academy of Sciences (IC), and the Royal Society of Chemistry.

In 2014 visible light induced PET-RAFT using Ir(ppy)_3_ as photocatalyst was reported.^107^ Since then zinc tetraphenylporphyrin (ZnTPP) was frequently used as metal-based photo catalyst in visible light induced PET-RAFT.^36,114–118^ Very recently Wanasinghe *et al.*^116^ used both Ir(ppy)_3_ and ZnTPP to analyze bulk swelling ratios and homogeneity of polymer networks, whereby better results were reported using ZnTPP. Corrigan *et al.*^119^ used ZnTPP as photoredox catalyst for a PET-RAFT polymerization at low-intensity yellow light irradiation, ambient temperatures, and in an open reaction vessel in the presence of oxygen. Also by using ZnTPP Shanmugam *et al.*^120^ polymerized styrene, (meth)acrylates, and (meth)acrylamides using various visible light wavelengths between 435–655 nm to activate the trithiocarbonate compounds. A variety of wavelengths has been used in combination with ZnTPP as ZnTPP has serval absorption maxima at 422 nm, 520 nm, 570 nm, and 600 nm (Fig. 3a). Shanmugam *et al.*^120^ used 2-(*n*-butyltrithiocarbonate)-propionic acid (BTPA) as trithiocarbonate to analyze the polymerization rates upon tuning the light wavelength. This study showed that the polymerization with yellow light was the fastest and the polymerization speed decreased from yellow > green > orange > red > blue light whereby the emission of the yellow lamp was centered on the maximum absorption at 570 nm. The group of Pester^109^ used ZnTPP for an oxygen tolerant, surface initiated PET-RAFT (SI-PET-RAFT) under visible light (405 or 590 nm, see Fig. 3b). In this study two different RAFT agents: 2-(dodecylthiocarbonothioylthio)-2-methylpropanoic acid (DDMAT) and 4-cyano-4-(phenylcarbonothioylthio)pentanoic acid (CPADB) were investigated. A higher monomer tolerance for CPADB-functionalized surfaces in combination with the photo catalyst Ir(ppy)_3_, showing a strong redox-potential, was described. Furthermore, the synthesis of block copolymers was demonstrated. The group of Pan^121^ also used the RAFT agent CPADB for a visible light induced PET-RAFT and demonstrated a temporal control over the polymerization and polymer formation by switching the light on and off (Fig. 3c). Additionally, the effects of catalyst concentration and light intensity on the polymerization rate were analyzed showing a gradual increase of monomer conversion with catalyst concentration and faster polymerization rate with increasing light intensity. Applying blue light irradiation (460 nm, irradiation time 24 h) Yeow *et al.*^122^ used a ruthenium-based photoredox catalyst (Ru(bpy)_3_Cl_2_) to activate a photoinduced PET-RAFT.

But not only metal-based catalysts are suitable for PET-RAFT induced by visible light. Recently, organic catalysts such as Eosin Y^123,124^ and fluorescein^125^ were applied to initiate RET-RAFT polymerizations. Using organic catalysts for a SI-PET-RAFT Kuzmyn *et al.*^124^ prepared antifouling polymer brushes on gold surfaces using three different monomers, namely oligo(ethylene glycol) methyl ether methacrylate (MeOEGMA), carboxybetaine methacrylamide (CBMA), and *N*-(2-hydroxypropyl) methacrylamide (HPMA). In 2016 Shanmugam *et al.*^113^ used Bacteriochlorophyll a as organic photoredox catalyst and demonstrated the first PET-RAFT under near-infrared/far-red irradiation (850 nm und 780 nm). Very recently Bellotti and Simounutti^126^ summarized theoretical basics, as well as industrial applications for PET-RAFT.

Unlike the classical RAFT or the PET-RAFT, the iniferter initiated polymerization does not require any additional catalyst which makes it an interesting candidate for polymerization in confined space. This might be an advantage for nanopore functionalization as nanopore accessibility to reagent might be an essential factor for polymerization control. Based on the visible light irradiation of trithiocarbonates (*e.g.* RAFT agents) the polymerization is initiated. Trithiocarbonate compounds possesses thiocarbonyl (C=S) groups. The absorption characteristics of thiocarbonyl-groups are slightly shifted to longer wavelengths, as compared to the absorption of carbonyl (C=O) groups. Thiocarbonyl groups show an absorption band in UV range at approximately 320 nm (spin allowed) and a second absorption band in the visible light range between 400–550 nm (spin-forbidden). Because of the absorption band at approximately 400–550 nm, compounds with thiocarbonyl groups, for example trithiocarbonate, react to visible light irradiation.^29^

**Fig. 4:**
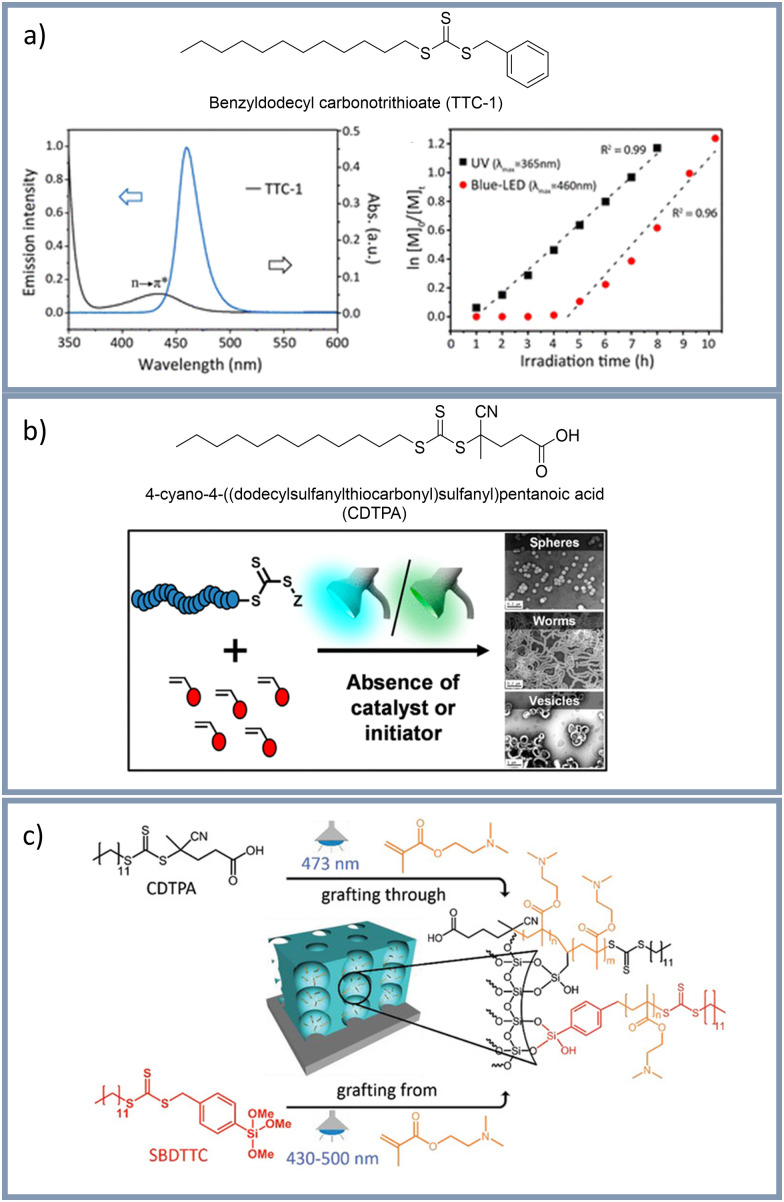
(a) Visible light induced photo-controlled radical polymerization using the Iniferter TTC-1 for the first time in absence of exogenous photoinitiators or catalysts by the group of Qiao.^29^ Reprinted with the permission from *Macromolecules*, 2015, **48**, 3864–3872.^29^ Copyright 2015 American Chemical Society (b) Synthesis of complex polymeric architectures using CDTPA as Iniferter under blue (460 nm) and green (530 nm) light irradiation by the group of Boyer.^30^ Reprinted with the permission from *ACS Macro Lett.*, 2016, **5**, 558–564.^30^ Copyright 2016 American Chemical Society. (c) Mesopore functionalization using CDTPA and SBDTTC as iniferter under visible light irradiation by our group.^130^ Reprinted with the permission from *Adv. Funct. Mater.*, 2021, **31**, 2009732.^130^ Copyright 2021 John Wiley & Sons, Inc.

For the first time, in 2015 the group of Qiao^29^ used the iniferter benzyldodecyl carbonotrithioate (TTC-1) for a light-triggered radical polymerization *via* visible light irradiation at approximately 460 nm in the absence of exogenous photoinitiators or catalysts (Fig. 4a). In this study the synthesis of well-defined poly(acrylamides) and poly(acrylates) was demonstrated. Furthermore, Qiao *et al.* showed that thermal induction of the polymerization could be excluded although the applied LED's heated the reaction mixture. In another example, as well from 2015, the group of Qiao^127^ used trithiocarbonate for a photo-controlled radical polymerization under visible light (460 nm) to generate cross-liked star polymers. Furthermore, Rubens *et al.*^25^ used a blue light source to initiate a polymerization, which was also controlled by trithiocarbonate. This study demonstrated full monomer conversion within one hour or even shorter reaction time. Therefore, no notable degradation of the trithiocarbonate was observed. The group of Boyer^30^ reported a photo-controlled radical polymerization using polymerization-induced self-assembly with 4-cyano-4-((dodecylsulfanylthiocarbonyl)sulfanyl)pentanoic acid (CDTPA) as iniferter under blue (460 nm) and green (530 nm) light irradiation (Fig. 4b). Probably due to differing degrees of polymerization control using two different light wavelengths, block-copolymer nanoparticles with different morphologies were obtained. In another example CDTPA was also used to synthesize polymeric nanomaterials with various morphologies using visible light irradiation and the organic photoredox catalyst PTH.^121^

Despite the recent dynamic developments in visible light induced RAFT including PET-RAFT and iniferter initiated polymerizations in solution and on planar surfaces examples on nanopore functionalization using visible light induced RAFT remain scarce up to date: The group of Wei^128^ used 4-cyano-4-(ethylsulfanylthiocarbonylsulfanyl)pentanoic acid (CEP) for RAFT polymerizations under 480 nm light to functionalized mesoporous silica nanoparticles with zwitterionic polymers. Recently, the first PET-RAFT in mesopores was reported by Joshi and Nebhani.^129^ After synthesizing mesoporous silica particles with an in-built RAFT agent, a visible light induced PET-RAFT was performed using a 50 W LED and Eosin Y as photo catalyst. This PET-RAFT in mesopores was achieved by stepwise co-condensation, using different hydrophobic and hydrophilic monomers. Our research group recently reported an asymmetric mesopore functionalization using a visible light induced PET-RAFT with ZnTPP as photo catalyst.^130^ In another example of our research group a layer-selective functionalization of mesoporous double layered films using the iniferter 4-(*N*,*N*-diethyldithiocarbamoylmethyl)benzoic acid (BDC) under UV-light irradiation was demonstrated.^131^ A visible light induced mesopore functionalization was also performed using two different iniferter approaches: Using the iniferter *S-p*-trimeth-oxysilylbenzyl-*S*′-dodecyltrithiocarbonate (SBDTTC) for a grafting from approach and CDTPA for a grafting through approach (Fig. 4c).^132^ Using these two visible light sensitive iniferters in combination with localized surface plasmon resonance from alloy Ag/Au nanoparticles as optical near field method 3D local polymer functionalization was demonstrated.^132^

## Ionic and other controlled polymerizations initiated by visible light

**Fig. 5:**
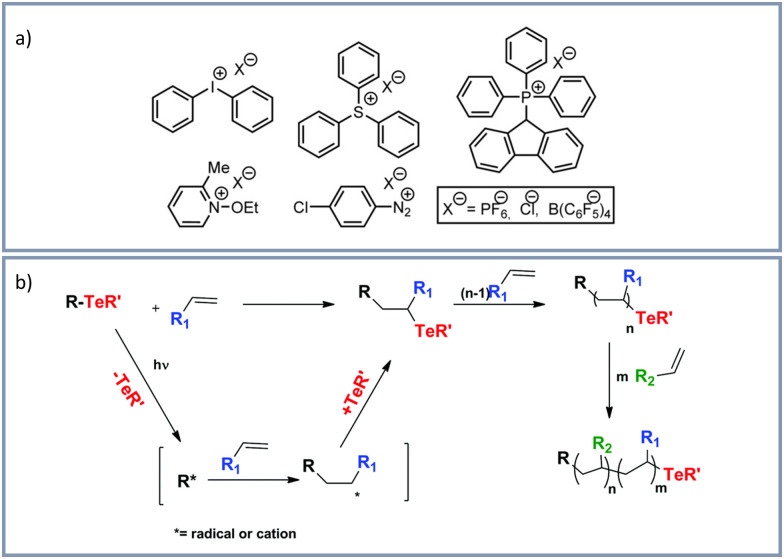
(a) Typical onium salts for photoinitated cationic polymerizations by Michaudel *et al.*^133^ Reprinted with the permission from *Angew. Chem.*, 2017, **129**, 9798–9808.^133^ Copyright 2017 Wiley-VCH Verlag GmbH & Co. KGaA, Weinheim. (b) Photoinduced organotellurium-mediated polymerization by Kaya *et al.*^138^ Reprinted with the permission from *Polym. Chem.*, 2018, **9**, 5639–5643.^138^ Copyright 2018 Royal Society of Chemistry.

Many examples on visible light induced cationic polymerizations have been reported. One reason for this is probably related to the tolerance of ambient oxygen and water. Most of the reported visible light induced cationic polymerizations are based on onium salt initiators.^133,134^ Examples of typical onium salts for photoinitiated cationic polymerizations are shown in Fig. 5a.^133^ Mechanistically this polymerization is based on the decay of the excited state of the onium salt after photoexcitation. Thereby, both, a homolytic and a heterolytic cleavage of the onium salt is possible, whereby highly reactive aryl cations and aryliodine cation radicals are generated. For more information on the mechanism of photoinduced cationic polymerization and the mechanism we refer to the highlight-article “The Discovery and Development of Onium Salt Cationic Photoinitiators” from Crivello in 1999.^134^

Regarding reported examples on cationic salt-based polymerizations, Yilmaz *et al.*^135^ performed a free radical promoted cationic polymerization under irradiation of visible light from 430–490 nm by using thioxanthone-fluorene carboxylic acid or thioxanthone-carbazole as photocatalysts and diphenyliodonium hexafluorophosphate or silver hexa-fluorophosphate as cationic salts. Sari *et al.*^136^ described a surface initiated free radical promoted cationic polymerization on tetrakis(2,4,6-trimethylbenzoyl)silane (TTBS) at room temperature and visible light (>400 nm) in presence of the onium salts diphenyliodonium hexafluorophosphate and triphenylsulfonium hexafluorophosphate. Also in the presence of onium salts Yilmaz *et al.*^137^ reported a visible light (>400 nm) induced cationic polymerization by fullerene sensitization. Kaya *et al.*^138^ used organotellurium compounds in the presence of diphenyliodonium hexafluorophosphate for polymerizations initiated by visible light and sunlight (organotellurium-mediated polymerization, Fig. 5b). Crivello^139^ reported a visible light induced cationic polymerization of epoxides with a titanium-complex free radical photoinitiator and diaryliodonium salts. Sangermano and coworkers^140^ performed cationic polymerizations using different onium salt photoinitiators. Kerem *et al.*^141^ used sulfonium salt photoinitiators. For a cationic ring-opening photo-polymerization of epoxide monomers and epoxide functional oligomers an efficient three-component visible light sensitive photoinitiator system was developed. The use of camphorquinone in combination with a benzyl alcohol generates radicals by visible light absorbance.^142^ Combining free radical and cationic polymerizations of vinyl monomers and cyclic ethers, initiated by visible light (400–500 nm), conjugated microporous polymeric networks containing thioxanthone groups were obtained.^143^

**Fig. 6:**
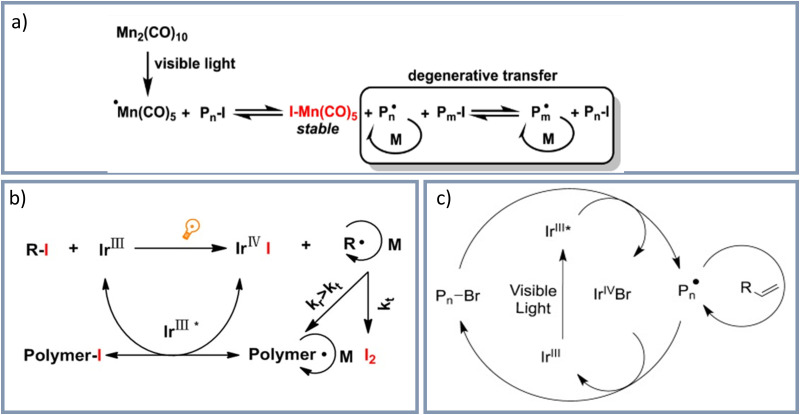
(a) Mechanism of photo induced ITP with Mn_2_(CO)_10_/alkyl iodide.^151^ Reprinted with permission from *Polym. Int.*, 2016, **65**, 1001–1014.^151^ Copyright 2016 John Wiley & Sons, Inc. (b) Visible-light induced ITP with Ir(iii) complex.^152^ Reprinted with permission from *J. Polym. Sci., Part A: Polym. Chem.*, 2014, **52**, 3283–3291.^152^ Copyright 2014 John Wiley & Sons, Inc. (c) Suggested mechanisms of the visible light induced polymerization using an Ir-based photoredox catalyst.^153^ Reprinted with permission from *Angew. Chem., Int. Ed.*, 2012, **51**, 8850–8853.^153^ Copyright 2021 John Wiley & Sons, Inc.

Besides these examples of cationic polymerizations, combined with free radical photoinitiators, it is also possible to combine free radical promoted cationic polymerization with controlled radical polymerizations: For example, the group of Yagci^144^ used Mn_2_(CO)_10_ as radical source and synthesized block copolymers by the combination from ATRP with visible light induced free radical promoted cationic polymerization. In a further example, the group of Yagci^145^ synthesized amphiphilic hyperbranched macromolecular structures in presence of Mn_2_(CO)_10_ by using the visible light induced self-condensing vinyl copolymerization of three methacrylates. Mn_2_(CO)_10_ for visible light induced polymerizations was not only applied in solution, furthermore surface functionalization was achieved: Xiong *et al.*^146^ described a visible light induced surface grafting polymerization with the use of Mn_2_(CO)_10_ on gold surfaces. The polymer-film thickness was controlled by variation of the irradiation time. The group of Chen^147^ reported the surface functionalization of poly(dimethylsiloxane) by visible light induced polymerization with Mn_2_(CO)_10_. Combining a visible light-induced free radical polymerization with a ROMP and hydrobromination the group of Yagci^148,149^ prepared a copolymer by grafting from using Mn_2_(CO)_10_ and visible light irradiation. Mn_2_(CO)_10_ as photo catalyst was also used for a visible light induced iodine transfer polymerizations (ITP) at 463 nm by Koumura *et al.*^150^ ITP is an controlled radical polymerization, which involves vinyl monomer, a conventional initiator and an iodinated chain transfer agent.^151^ The general mechanism of the polymerization using Mn_2_(CO)_10_ is shown in Fig. 6a.^151^ The applications of visible light induced photo initiation based on Mn_2_(CO)_10_ was summarized by the group of Yagci in 2016.^151^ Furthermore, visible-light induced ITP have be realized using an Ir(iii) complex (Fig. 6b).^152^ Fors and Hawker^153^ portrayed a visible light induced living radical polymerization with ppm amounts of the photocatalyst *fac*-[Ir(ppy)_3_]. The suggested mechanism of the polymerization is shown in Fig. 6c.

These examples represent a selection among the manifold studies on visible light induced polymerizations based on cationic polymerization, whereby especially polymerizations based on onium salts or Mn_2_(CO)_10_ are frequently used. Most of the studies on this type of polymerization were performed at wavelengths below 600 nm and in solution. Going towards red light irradiation, the group of Fouassier reported the development of cationic polymerizations and showed the possibility to use various wavelengths, including red light irradiation.^154^ Despite the plenty examples on visible light induced cationic polymerizations in solution and even on surfaces, studies on polymer functionalization of nanoporous materials using visible light induced cationic polymerizations remain unreached (Fig. 8).

## Visible light induced ROMP

**Fig. 7:**
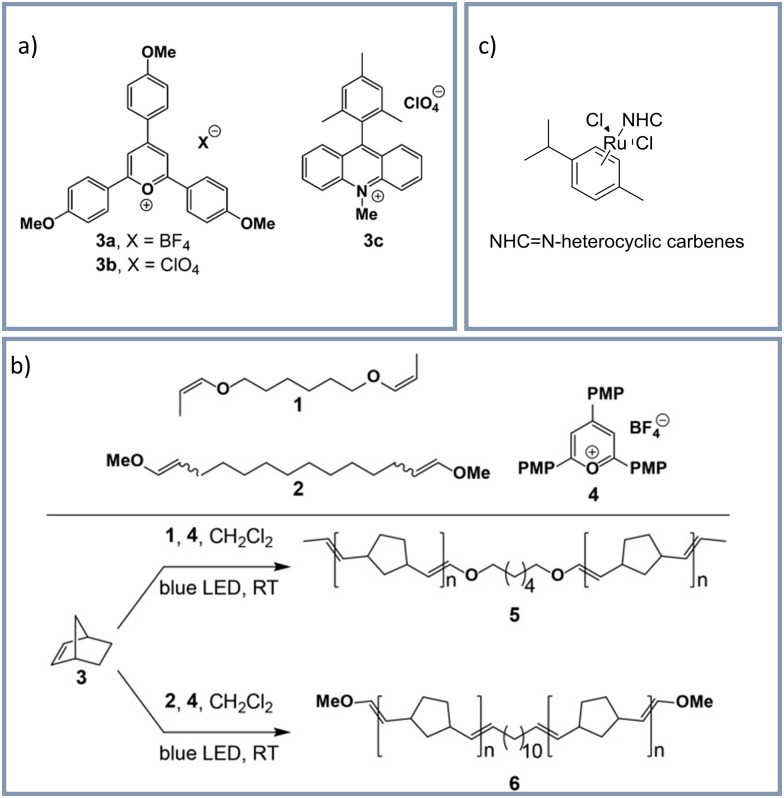
(a) Photoredox mediators for metal free ROMP under blue light irradiation by the group of Boydston.^156^ Reprinted with the permission from *J. Am. Chem. Soc.*, 2015, **137**, 1400–1403. Copyright 2015 American Chemical Society. (b) Photo catalyzed ROMP by the group of Boydston^157^ Reprinted with the permission from *J. Polym. Sci., Part A: Polym. Chem.*, 2017, **55**, 2977–2982.^157^ Copyright 2017 John Wiley & Sons, Inc. (c) General structure of visible light catalysts for ROMP.^160^ Reprinted with permission from *Synlett*, 2016, **27**, 203–214.^160^ Copyright 2016, Rights Managed by Georg Thieme Verlag KG.

Besides visible-light induced cationic polymerizations and ITP visible-light induced ROMP represents another, complementary polymerization technique beyond radical polymerization. Only a few examples on visible light induced ROMP have been demonstrated. In general, visible light induced ROMP are still relatively scarce, and so far limited to solution polymerizations. In the traditional ROMP metal-complexes, *e.g.* Ru-, W-, or Mo-alkylidene complexes, are used as catalysts.^155^ Also organocatalyzed ROMPs have been reported, which use vinyl ethers as initiators^156,157^ In 2015 the group of Boydston^156^ reported the first metal free ROMP using blue-light emitting LEDs (450–480 nm). The used photoredox mediators compatible with this blue-light LED irradiation are shown in Fig. 7a. Theunissen *et al.*^158^ published a ROMP and photolithographic olefin metathesis polymerization (PLOMP) using blue light. Using divinyl ether initiators and a photoredox catalyst to polymerize norbornene upon 450–480 nm light irradiation, the group of Boydston^157^ successfully performed a visible light induced ROMP. Fig. 7b shows the used divinyl ethers (1 and 2) and the pyrylium photocatalyst (4). Recently Eivig *et al.* reported ROMP under 420 nm irradiation as suitable for 3D applications.^159^ Catalysts with the general structure shown in Fig. 7c, absorb at 450 nm and can also be used for visible light control of ROMP.^160^

Examples on nanoporous material functionalization using classical ROMP remain scarce and examples on visible light induced ROMP in nanopores have not yet been reported. For classical ROMP Mohite *et al.*^161^ polymerized free norbornene using ROMP in nanoporous silica nanoparticles, whereby it was bound to surface-grafted norbornene by using a crosslinking mechanism. Our research group reported on ROMP being used to functionalize nanopores with 5-norbornene-2-carboxylic acid pentafluorophenyl ester^162^ as well as with spiropyran and spirooxazine substituted polynorbornene homopolymers.^163^ Plenio and colleagues^164^ showed an alternative approach when covalently binding the catalyst to a silica nanoparticle surface followed by a SI-ROMP in presence of monomer in solution.

## Conclusions

**Fig. 8:**
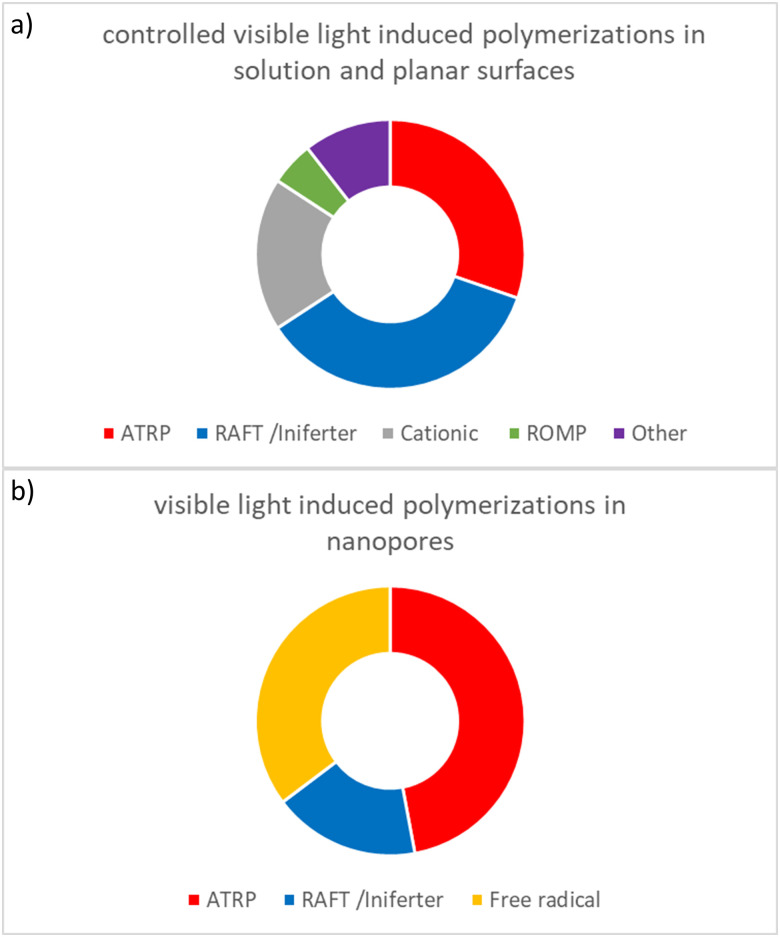
Comparative summary of (a) controlled visible light induced polymerizations in solution and on planar surfaces, mentioned in this article (in total 76 references) and (b) first examples on visible light induced polymerizations in nanopores as cited in this article (in total 17 references).

In summary, research on visible light induced polymerizations has been dynamically evolving over the last years. In particular, many examples of visible-light induced cationic polymerizations, ATRP, and RAFT in solution and on planar surfaces have been reported mainly within the last two decades (Fig. 8a). Main research activities within the last years have been focused on broadening the wavelengths range to cover the entire range of visible light, *e.g.* using metal photo catalysts. Subsequently, the number of reported studies on visible light induced polymerization increased and the wavelength range was shifted significantly into the visible wavelength range. Furthermore, the reduction of these metal photo catalysts in the reaction was strongly investigated resulting in synthesis strategies allowing metal-free visible light controlled polymerizations with increasing number of reported studies in recent years. Although the number of examples is still limited, visible light induced controlled radical polymerizations such as visible-light induced ATRP, visible-light induced PET-RAFT, dye-sensitized polymerizations, and visible light induced iniferter initiated polymerizations were successfully transferred to nanopore functionalization (Fig. 8b). ATRP is the most frequently used visible light induced polymerization in nanopores, with a contribution of 47% of the cited references in this review. Nevertheless, visible light induced PET-RAFT is especially significant when considering that only very recently in 2022 the first examples of PET-RAFT in nanopores was reported. The differences between the polymerization techniques in solution and on planar surfaces as compared to nanopores as well clearly demonstrates the challenges in nanopore visible-light polymerization with respect to ionic and ROMP polymerization. Visible light induced controlled radical polymerizations are envisioned to be of special interest with respect to automated and advanced material fabrication and functionalization strategies. Here in particular the potential enabling high local control on polymer placement in nanoporous materials is of interest. Mechanistically, the question of how confinement, local concentration variations as well as nanopore accessibility and transport influence such visible light induced polymerization still bares many open questions. In this context recent work on confinement controlled catalysis^165–167^ together with theoretical work on ion concentration profiles along nanopore cross sections^14^ points towards deeper understanding of confinement influences on reactions and represents a first starting point towards a rational confinement-controlled design. However, compared to the many examples in solution and even on planar surfaces, the development of visible light induced polymerizations for nanopore functionalization is still in its infancy (Fig. 8) but inherits great potential especially for automated polymer writing, highly precise polymer placement, or additive manufacturing.

## Author contributions

Claire Förster: writing original draft, visualization, investigation. Annette-Andrieu-Brunsen: acquisition of funding, supervision and assistance with manuscript writing.

## Conflicts of interest

There are no conflicts to declare.

## Supplementary Material
